# Real-time estimation of biomass and specific growth rate in physiologically variable recombinant fed-batch processes

**DOI:** 10.1007/s00449-012-0848-4

**Published:** 2012-11-23

**Authors:** Patrick Wechselberger, Patrick Sagmeister, Christoph Herwig

**Affiliations:** Research Area Biochemical Engineering, Institute of Chemical Engineering, Vienna University of Technology, Gumpendorfer Straße 1a, 1060 Vienna, Austria

**Keywords:** Recombinant protein production, Process model, Soft sensor, Real-time biomass quantification, Process analytical technology (PAT)

## Abstract

The real-time measurement of biomass has been addressed since many years. The quantification of biomass in the induction phase of a recombinant bioprocess is not straight forward, since biological burden, caused by protein expression, can have a significant impact on the cell morphology and physiology. This variability potentially leads to poor generalization of the biomass estimation, hence is a very important issue in the dynamic field of process development with frequently changing processes and producer lines. We want to present a method to quantify “biomass” in real-time which avoids off-line sampling and the need for representative training data sets. This generally applicable soft-sensor, based on first principles, was used for the quantification of biomass in induced recombinant fed-batch processes. Results were compared with “state of the art” methods to estimate the biomass concentration and the specific growth rate* µ*. Gross errors such as wrong stoichiometric assumptions or sensor failure were detected automatically. This method allows for variable model coefficients such as yields in contrast to other process models, hence does not require prior experiments. It can be easily adapted to a different growth stoichiometry; hence the method provides good generalization, also for induced culture mode. This approach estimates the biomass (or anabolic bioconversion) in induced fed-batch cultures in real-time and provides this key variable for process development for control purposes.

## Introduction

### Motivation

One of the key tasks in process development is maximization of space–time yield of the product while maintaining a previously defined product quality related attributes. Even though recombinant protein production is not strictly growth related as products of primary metabolism (e.g. ethanol), it is usually tied to the physiological state of the culture [1, 2]. Fed-batch process mode provides good metabolic control over the cell metabolism, since the availability of the limiting substrate is governed by the feeding profile. Hence, it is an important goal of process development to come up with a feeding strategy beneficial for both product yield and product quality. Here, a key variable is the biomass concentration which is required for further calculation of variables describing the metabolic state of the culture such as specific rates (e.g. the specific growth rate) and yields. The reference method for biomass is gravimetric determination of biomass dry weight, which is typically defined as the insoluble fraction of the culture broth after removal of water (e.g. by drying at 105 °C). Conventionally, this is determined by off-line quantification, which is time-consuming and also comes with high operator dependent measurement error. Dry cell weight determination accounts for the insoluble fraction of mass in biomass, while it would physiologically more relevant to quantify the cells actually taking part in the bioreaction [3]. Especially in induced cultures there is variation in cell morphology [4], energy metabolism [5] and macroscopic composition of the cells; hence quantification of “biomass” or similar variables is not straight forward [6]. But most importantly there has to be a clear link to product quality or productivity, which is also process or product dependant. Furthermore, the variable should become available in real-time for control strategies and also to speed up process development using parallel processing [7] and automation of experiments [8, 9].

### Differentiation from existing methods

#### Hard-type sensors

Various sensors for biomass using optical methods (turbidity, near infrared, NADH) or radio impedance (capacitance) are available for the in-line quantification of the biomass concentration [6, 10–12]. Since determination of biomass dry weight is not feasible directly (in situ) in the bioreactor for obvious practical reasons, this variable has to be quantified by relation to physical characteristics or morphological properties of the cells (e.g. electrical impedance or light scattering properties). However, changes in the physiological state of the culture are often accompanied by physiochemical or morphological changes in the cell population, potentially distorting established correlations. Different calibrations can be necessary for different process modes, media composition, and physiological states of the culture and also for different strains. Model maintenance or very good generalization (however, this often comes at the cost of estimation accuracy) is required to account for variability of the relation between the probe response and the actual dry cell weight, since the relation can vary, e.g., dependent on the media constituents and other process characteristics [13, 14], especially in process development with constantly changing process environments, culture conditions, strains, etc. it can be misleading to use these hard-type sensors as a sole quantification of biomass in process development. On the other hand this can be very useful to acquire interesting real-time information on the physiological state of the culture [15]; however, it is often not possible to determine the source of variability (e.g. morphological change or increase in biomass) from the real-time signal only. Thus, this should be seen as an auxiliary tool together with complementary methods (e.g. flow cytometry).

#### Soft-type sensors

Soft-type sensors make use of easily accessible items such as carbon dioxide and oxygen concentrations in the off-gas stream (using well established methods such as infrared for CO_2_ or paramagnetic principle for O_2_, respectively) to indirectly quantify less “easy” items such as the total biomass concentration [16]. There are two main competing approaches, empiric or data-driven methods and mechanistic models based on fundamental knowledge.

One school of thought came up with data-driven methods such as PCA, ANNs, PCR, PLSR [17–20]. No prior knowledge is required, but representative training data sets for the modeling problem to estimate model coefficients or to train weights (ANNs) are an imperative. Hence, these methods are useful if the process conditions do not vary too much (e.g. in production) and they come with constrained generic applicability as a soft sensor in process development [16].

A more direct approach to generate knowledge is using mechanistic models, which try to describe the system in question by fundamental principles (e.g. chemical or physical) on the interaction between process variables [21, 22]. The advantage of mechanistic models is also a drawback; detailed knowledge on the mechanistic of the process is not always available. In biological processes setting up mechanistic models is especially challenging due the great complexity of the living cell. Hence, one should avoid extensive use of prior knowledge or frequent re-fitting of parameters, as there would be no gain in the ease of use compared to hard-type sensors.

#### Soft-type sensors based on first principles

First principles are generally valid (e.g. the law of conservation, or equations of state for vapor–liquid equilibriums); hence, this is ideal for the dynamic field of process development, since these can be easily adapted to a new problem. For this reason first principles should be used whenever possible and calibration/training should be avoided. First principles require little prior knowledge, mostly items which are quantified anyways, e.g. growth stoichiometry and quantification of in-going and out-going mass streams (substrate, oxygen, carbon dioxide, etc.).

Here, a minimalistic mechanistic model relying on first principles rather than prior knowledge is suggested for extraction of information, consistency check and estimation of unknown items in physiologically variable cultures. While the basic idea of this approach has been published decades ago [21, 23, 24], these ideas are interesting in the context of modern industrial recombinant bioprocesses, due to their ease of use and general applicability. The model approach is black box and unsegregated for sake of real-time capability. For general applicability the model is biochemically structured, based on macroscopic mass balances; hence, extensive use of model parameters which have to be experimentally determined was avoided. Ideally only natural constants, direct measurements and first principles should be used. Using enough constraints such as elemental balances, estimation of parameters such as yield coefficients from previous experiments can be avoided. Furthermore, redundancy should be applicable (the equation system is over determined), so that the consistency of the estimation can be verified. This is very useful to detect gross errors such as wrong stoichiometric assumptions or sensor failure.

#### Context overview

Induction of recombinant protein can have significant impact on the energy metabolism of cells, e.g. *E. coli* cultures this can result in partial cell lysis [25, 26] and unspecific release of proteins, carbohydrates and other building blocks to the supernatant. Alterations in the energy metabolism can also result in variations of yield coefficients. For this reason, models making use of fixed yields coefficients are wrong as soon as there is unaccounted variations in these coefficients. Some authors [27] came up with the idea of supplementing mechanistic models with data-driven methods such as neural networks to tackle that problem, but this does not eliminate the need for representative training data sets in the first place. Another approach to deal with model uncertainties such as poor knowledge of model coefficients is Kalman filters. Deviations of the process model can be mitigated by incorporation of off-line samples [28], however this does not eliminate off-line sampling and still requires prior knowledge on model coefficients. We want to avoid off-line sampling and the need for representative training data sets at all, using an elemental balancing approach, which relies on first principles only. While the basics for this approach were already published [16, 29], this contribution focuses on quantification of the biomass in the induction phase of fed-batch processes in red biotechnology, which is a key variable for process optimization as discussed above.

**Fig. 1:**
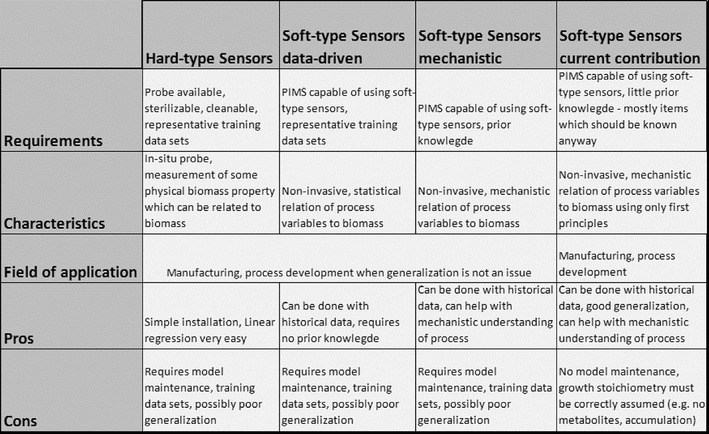
Overview on approaches to quantify biomass in real-time

Figure 1 shows an overview on approaches to quantify biomass in real-time discussed in the previous chapters.

### Goals

- We demonstrate an approach for the estimation of biomass concentration and specific growth rate for processes with variable cell metabolism and morphology and evaluate its applicability. For instance, this can be during induction phase of recombinant processes, where the quantification of biomass is a challenge. The method avoids off-line sampling and the need for representative training data sets.
- Different methods to calculate the specific growth rate in real-time, which is a key variable for process optimization and is typically calculated from off-line biomass concentrations, are compared, including a soft-sensor approach based on cumulative elemental balancing, a Luedeking–Piret-type (fixed yield) approach, based on off-gas rates and a hard-type capacitance probe.
- The approach should be useful for control and be available as a key variable for process development.
- Consistency check: gross errors such as wrong stoichiometric assumptions or sensor failure should be detected automatically.

## Materials and methods

### Culture

#### *Pichia pastoris* as eukaryotic microbial model system

The *Pichia pastoris* strain KM71H expresses the horseradish peroxidase isoenzyme C1A (HRP). The strain was of MutS (methanol utilization slow) phenotype and HRP was secreted into the fermentation broth. Media were prepared according to [30]. After shaking flask preculture, a batch cultivation was initiated, followed by a fed-batch on glycerol to increase the biomass and induction phase on methanol employing a feeding strategy according to [28].

#### *E. coli* as prokaryotic microbial model system

A recombinant K12 *E. coli* strain with alkaline phosphatase on a rhamnose inducible promoter was used for the verification runs with stoichiometrically defined media [25]. A shaking flask preculture (100 ml for inoculation of 6 L batch medium, in 1 L shaking flask with baffles) was inoculated from frozen stocks and was used to inoculate the bioreactor. Culture conditions were pH = 7, temperature = 35 °C and DO_2_ > 20 %. After a batch phase, which was detected by a drastic drop in the CO_2_ off-gas signal and an increase in dissolved oxygen (DO_2_), an exponential fed-batch with a specific growth rate of 0.15 (h^−1^) was initiated. Equations (1) and (2) were used to calculate the feed profile for the exponential fed-batch. The specific growth rate before induction was set prior to the experiment, while constants such as the feed concentration (*S*_0_), density (*ρ*_feed_), the initial volume (*V*_0_) and initial biomass concentration *X*_0_ were measured. The biomass yield (*Y*_x/s_) was determined in prior experiments. The molecular weight of substrate and biomass (*M*_S_, *M*_X_) can be found in the literature or measured by a CHON analyzer in prior experiments. The exponential phase was followed by an induction phase with linear feeding, which was adjusted to an initial specific growth rate of different percentages of the exponential phase growth rate, 100, 70 and 40 % or 0.15, 0.1 or 0.06 (h^−1^), respectively.

Feedrate in exponential fedbatch

1 $$ F_{(t)} = F_{0} *\,e^{k\;*\;t} . $$

Initial feed rate in exponential fedbatch

2 $$ F_{0} = \frac{{k*X_{0} *M_{\text{s}} *\rho_{\text{feed}}*V_{0} }}{{S_{0} *Y_{{{{\text{x}} \mathord{\left/ {\vphantom {{\text{x}} {\text{s}}}} \right. \kern-\nulldelimiterspace} {\text{s}}}}} *M_{\text{X}} }}. $$

#### Biomass

Biomass concentrations were quantified by gravimetric measurement after drying for 72 h at 105 °C. Samples were centrifuged (5,000 rpm, 10 min) and the pellet was washed twice with distilled water to get rid of salts.

#### Substrate and small metabolites

Substrate and small metabolite concentrations were quantified using an HPLC method (Supelcogel C-610, Sigma Aldrich, flowrate: 0.5 ml/min, eluent: 0.1 % H_3_PO_4_/NaN_3_, 30 °C, RI detector).

#### Protein determination: BCA

Extracellular protein concentrations were measured using the Bicinchoninic Acid Kit for Protein Determination (Sigma, BCA1-1KT). Bovine Serum Albumin (BSA) was used as a standard. The limit of quantification (blank + 9 standard deviations) was determined to be 0.151 (g/l) with a residual standard deviation of 0.008 (g/l).

### Bioreactor setup and on-line analytics

#### Bioreactor

Two stainless steel bioreactors with working volumes of 10 and 20 L were used (Infors, Bottmingen, Switzerland). The systems come with a controller unit, which was used to adjust the process parameters: pH, temperature, aeration, reactor pressure and stirrer speed. Dissolved oxygen (DO_2_) was controlled >20 % using a step controlled with reactor pressure, stirrer speed and air flow as manipulated variable. The pH was controlled using an integrated digital peristaltic pump and NH_4_OH as a base. Air was filtered by a membrane-type filter and dispensed by a ring sparger. The culture vessel was sterilized at 121 °C for 20 min by in situ steam sterilization prior to inoculation.

#### Off-gas analysis

CO_2_ and O_2_ in the off-gas were quantified by a gas analyzer (Servomex, UK; M. Müller AG, Switzerland), using infrared and paramagnetic principle, respectively. Air flow was quantified by a mass flow controller (Vögtlin, Aesch, Switzerland).

#### Capacitance probe

An annular-type probe (Aber Instruments, Aberystwyth, Wales, UK) was used to measure capacitance during the fermentation. Capacitance values are calculated in real-time from the difference between two frequencies. At 1 MHz *E. coli* cells contribute to the capacitance while 10 MHz is the “background” depending on the medium, according to definitions of the supplier. The difference in capacitance relates to the viable cell concentration or more directly to intact biovolume, as only intact cells act as a capacitor [31].

#### Data management

For recording of process data the process information management system Lucullus from Biospectra (Schlieren, Switzerland) was used. This system was also used for closed loop control (feed bottle on balance).

### Setup of the soft sensor for estimation of biomass concentration and specific growth rate with variable growth stoichiometry

#### Conversion rates

Assuming oxidative metabolism, the bioreaction can be described according to Eq. 3. Although there are many different chemical reactions running in parallel in living cells, the conversion rates in Eq. 3 represent the overall summarized effect of all the different reactions.

Stoichiometric equations

3 $$ r_{s} \,\,{\text{CH}}_{{p{\text{H}}}} \,{\text{O}}_{{p{\text{O}}}} + r_{{{\text{O}}_{2} }} \,{\text{O}}_{2} + r_{N} \,{\text{NH}}_{3} \, \to r_{x} \,{\text{CH}}_{{z{\text{H}}}} \,{\text{O}}_{{z{\text{O}}}} \,{\text{N}}_{{z{\text{N}}}} + r_{{{\text{CO}}_{ 2} }} \,{\text{CO}}_{2} . $$

General material balance

4 $$ {\text{Input}} - {\text{output}} + {\text{conversion = accumulation}} . $$

The conversion rates in Eq. (4) for the species substrate (*S*), biomass (*X*), carbon dioxide (CO_2_), ammonia (N) as well as oxygen (O_2_) can be derived from the general form of the material balance [Eq. (4)].

In fed-batch mode the conversion rates can be calculated as follows:

Conversion rate for substrate

5 $$ r_{s} = \frac{d(s)}{dt} - \dot{S}_{\text{in}} + \dot{S}_{\text{out}} = - \frac{{F_{f,{\text{in}}} }}{{\rho_{feed} }}\,\,{S_{0}} . $$

In fed-batch mode, the outflow term $\dot{S}_{\text{out}}$ is zero and the accumulation term $\frac{d\left( s \right)}{dt}$ can be neglected, as long *μ* < *μ*_max_; hence, the conversion rate *r*_*s*_ is only dependent on the inflow term $\dot{S}_{\text{in}}$ which is calculated from the feed rate.

Conversion rate for biomass

6 $$ r_{x} = \frac{d\left( X \right)}{dt} - \dot{X}_{\text{in}} + \dot{X}_{\text{out}} = \frac{d\left( X \right)}{dt}. $$

Since there is no in- and outflow term, *r*_*x*_ is equal to the accumulation term $\frac{d\left( X \right)}{dt}$.

Conversion rate for carbon dioxide (=CER)

7 $$ r_{{\text{CO}}_{ 2}}  = {\text{CER}} =\frac{{\text{d (CO}}_{2} )}{{\text{d}}t}  -  {\text{C}}{\dot{\text{O}}}_{2,{\text{in}}} + {\text{C}}{\dot{\text{O}}}_{2,{\text{out}}}  = \frac{F_{a,{\text{in}}} }{V_{m} }(y_{{{\text{CO}}_{ 2} ,{\text{out}}}} {\text{Ra}}_{\text{inert}} - y_{{{\text{CO}}_{ 2} , {\text{in}}}} )\,*\,60. $$

Conversion rate for oxygen (=OUR)

8 $$ r_{{{\text{O}}_{ 2} }} = {\text{OUR}} =\frac{{d\left( {{\text{O}}_{2} } \right)}}{dt} - {\dot{\text{O}}}_{{ 2 , {\text{in}}}} + {\dot{\text{O}}}_{{ 2 , {\text{out}}}} = \frac{{F_{{a,{\text{in}}}} }}{{V_{m} }}\,\left( {y_{{{\text{O}}_{ 2} , {\text{out}}}} \,{\text{Ra}}_{\text{inert}} - y_{{{\text{O}}_{ 2} , {\text{in}}}} } \right)\,*\,60. $$

Inert gas ratio

9 $$ {\text{Ra}}_{\text{inert}} = \frac{{1 - y_{{{\text{O}}_{ 2} ,{\text{in}}}} - y_{{{\text{CO}}_{ 2} , {\text{in}}}} }}{{1 - y_{{{\text{O}}_{ 2} , {\text{out}}}} - y_{{{\text{CO}}_{ 2} , {\text{out}}}} - \frac{{y_{\text{wet}} }}{{y_{{{\text{O}}_{ 2} , {\text{in}}}} }}}}. $$

Due to the low solubility of O_2_ in the fermentation broth, $\frac{{d\left( {{\text{O}}_{ 2} } \right)}}{dt}$ can be neglected. The term $\frac{{d\left( {{\text{O}}_{ 2} } \right)}}{dt}$ can be also neglected, since the solubility of CO_2_ in the fermentation broth is mainly a function of temperature and pH, which are typically kept constant. Hence, the rates $r_{{{\text{CO}}_{ 2} }}$ and $r_{{{\text{O}}_{ 2} }}$ are dependent on the in- and outflow terms (Eqs. 7 and 8). $F_{{a,{\text{in}}}} ,\,y_{{{\text{CO}}_{ 2} , {\text{out}}}}$ and $y_{{{\text{CO}}_{ 2} , {\text{out}}}}$ are measured, while ${\text{Ra}}_{\text{inert}}$ (Eq. 9) depends on the dilution by water stripping describes the ratio between the in- and outflow term. $y_{\text{wet}}$ is the off-gas concentration of O_2_ without bioreaction and relates to the dilution by water stripping [32].

#### Specific rates and yields

Conversion rates are the basis for the computation of yields (Eq. 10). Specific rates are calculated according to Eq. (11). Specific growth rates using off-line or soft-sensor biomass concentrations were calculated according to Eq. (12). The Luedeking–Piret-type Eq. (13) (as found in the literature [33], assuming constant maintenance) can be reformed to calculate specific growth rates from off-gas rates only, since the yield can be eliminated from the equation as long it is assumed to be constant (the maintenance part does not vary). Specific growth rates based on total carbon or total oxygen were computed according to Eqs. (14) and (15). The total cumulated carbon dioxide $t{\text{CER}}$ is equal to the carbon dioxide rate ${\text{CER}}$ integrated with $\Updelta t$, plus an initial value $t{\text{CER}}_{\text{initial}}$. The specific growth rate is subsequently calculated by the fraction of ${\text{CER}}$ by $t{\text{CER}}$. Similarly specific growth rates based on the capacitance signal were calculated according to Eq. (16) by the fraction of the capacitance rate by the total capacitance.

Calculation of yields

10 $$ Y_{\frac{i}{j}} = \frac{{r_{i} }}{{r_{j} }}. $$

Calculation of specific rates

11 $$ q_{i} = \frac{{r_{i} }}{X}. $$

Calculation of the specific growth rate from biomass conversion rates

12 $$ \mu = \frac{{r_{x} }}{X}. $$

Luedeking–Piret-type equation, on the example of CO_2_

13 $$ {\text{CER}} = Y_{{{{{\text{CO}}_{ 2} } \mathord{\left/ {\vphantom {{{\text{CO}}_{ 2} } X}} \right. \kern-\nulldelimiterspace} X}}} \,*\,\,\mu \,*\,X. $$

Calculation of total cumulated off-gas rates, on the example of CO_2_

14 $$ t{\text{CER}} = {\text{CER}}*\Updelta t + t{\text{CER}}_{\text{initial}} . $$

Calculation of the specific growth rate from off-gas rates on the example of CO_2_

15 $$ \mu_{{t{\text{CER}}}} = \frac{{r_{x} \,*Y_{{{{{\text{CO}}_{ 2} } \mathord{\left/ {\vphantom {{{\text{CO}}_{ 2} } X}} \right. \kern-\nulldelimiterspace} X}}} }}{{X\,*Y_{{{{{\text{CO}}_{ 2} } \mathord{\left/ {\vphantom {{{\text{CO}}_{ 2} } X}} \right. \kern-\nulldelimiterspace} X}}} }} = \frac{\text{CER}}{{t{\text{CER}}}} $$

Calculation of the specific growth rate from the capacitance

16 $$ \mu_{\text{cap}} = \frac{{d\left( {{\text{Capaci}}\,{ \tan }\,{\text{ce}}} \right)}}{dt}\frac{1}{{{\text{Capaci}}\,{ \tan }\,{\text{ce}}}}. $$

#### Constraints

General form of constraints with *k* elemental balances

17 $$ \sum\limits_{i = 1}^{k} {r_{i} \,v_{i} = 0} . $$

Using the law of conservation, elemental balances (=*k*) can be imposed on every element of the bioreaction as constraints (Eq. 17). In which *r* is the rate vector and *v* is the vector of coefficients for each element. This is useful as a consistency check of the data and to calculate non-measured items. In this contribution two balances were used, the carbon (C) balance and the degree of reduction (DoR) balance. The degree of reduction balance can be set up with the following definition: *γ*_N_ = −3, *γ*_C_ = C = 4, $\gamma_{{{\text{O}}_{ 2} }}$ = O_2_ = −4, *γ*_H_ = 1. Other definitions would lead to the same results [34].

#### Sensitivities and error propagation

The random measurement error of off-gas analysis and gravimetric balances which are used as an input for this soft sensor are typically very low (relative error = 0.01–0.1 % propagated to the rates, using definitions according to supplier), hence can therefore be neglected. However, systematic errors such as miscalibration, sensor drift or the measurement error on the constants have to be considered. One percent deviation in feed concentration or density directly propagates as 1 % deviation of the substrate uptake rate. One percent deviation on the off-gas dilution by water (y_O2__wet) propagates up to 3 % on the oxygen uptake rate, depending on the measured oxygen concentration. Furthermore, the off-gas rates can be prone to miscalibration and sensor. As the soft sensor is directly based on these rates, the error on the estimated accumulated biomass (the difference between the current value and the start value) is supposed to be in the magnitude of a linear combination of these effects. Gross errors such as wrong stoichiometric assumptions (e.g.: oxidoreductive instead of oxidative growth) also contribute to these effects.

#### Consistency check

To evaluate the residuals on the rates when applying the constraints in “Constraints” and compare them with the expected residuals due to measurement error (“Sensitivities and error propagation”), a statistical test adapted from the literature [35] was applied to get a quantitative measure on the validity of the observed system. Equation (17) can be written in matrix form (Eq. 18):

Matrix form of constraints

18 $$ EW = 0. $$

*W* is the vector of the measured volumetric rates *r*.

For noisy data a residue vector $\varepsilon$ is added (Eq. 19):

Matrix form of constraints with residue vector

19 $$ E^{'} W = \varepsilon . $$

For each rate an expected error (by default 3 % error on each rate) is specified in the variance–covariance matrix Ψ of the rates and is assumed to be non-correlated (square with the errors for each rate in the diagonal). The result of the statistical test value *h* is calculated with Φ as the variance–covariance matrix of the residuals Eqs. (20) and (21). The hypothesis of not having any errors exceeding the expected error specified in Ψ is rejected if *h* is greater than a certain threshold value. This threshold value can be read from Chi-square distribution, which depends on the degree of redundancy of the equation system (or also the degree of freedom of the Chi-square distribution) and the significance level* α* (by default 0.95). The default* α* degree of redundancy of one (=estimation of one rate) results in a threshold of 3.84 for the statistical test value, which is exceeded if the current error is higher than the expected error (e.g. when gross errors such as wrong stoichiometric assumptions are present). The expected error was assumed to be 3 % error on each rate. An error of 3 % on each rate results in a deviation of about 10 % on the C- and DoR balance. The degree of redundancy of the equation system is equal to the rank of *E* if no conversion rates are estimated or to the rank of *R* if conversion rates are estimated.

Variance–covariance matrix

20 $$ \Upphi = E^{T} \,\Uppsi \,E. $$

Statistical test value

21 $$ h = \varepsilon^{T} \,\Upphi^{ - 1} \,\varepsilon . $$

#### Data reconciliation

A data reconciliation procedure according to [36] was applied. In addition to estimation of non-measured conversion rates, redundancy in the equation system can be also used to adjust the conversion rates to simultaneously close all elemental balances imposed in “Constraints”. The lumped residues of the equation system are distributed along the rates according the expected error for each rate. Using a least squares approach, the goal of reconciliation is to find a measurement error vector $\delta$ to calculate the reconciled vector $W_{\text{b}}$ (Eq. 22), hence the vector of the best estimates of the volumetric reaction rates to fit all constraints. The solution (Eq. 23) to this problem is adapted from the literature [37].

Calculation of the reconciled vector Eq. (22)

22 $$ W_{\text{b}} = W + \delta. $$

Calculation of the measurement error vector Eq. (23)

23 $$ \delta = \Uppsi \,E^{T} \,\Upphi^{ - 1} \,\varepsilon. $$

#### Estimation

For estimation of non-measured components the equation system from “Consistency check” is split into measured part $\left( {E_{{{\text{m}}\,}} \,\,W_{\text{m}} } \right)$ and a calculated part [$E_{\text{c}} \,W_{\text{c}}$; Eq. (24)].

Split of equations system

24 $$ EW = E_{\text{m}} \,W_{\text{m}} + E_{\text{c}} \,W_{\text{c}} = 0. $$

The law of conservation can be also applied on measured rates exclusively, but first the elemental matrix has to be stripped of relations with the calculated items, the resulting matrix is called the redundancy matrix *R* (Eqs. 25 and 26). If *R* contains zero columns, there is no way to express the measured rates independent from the non-measured rates. The rank of *R* is equal to degree of redundancy of the equation system.

Introduction of the redundancy matrix *R*

25 $$ RW_{\text{m}} = 0.$$

Calculation of the redundancy matrix *R*

26 $$ R = E_{\text{m}} - E_{\text{c}} \,E_{\text{c}}^{ - 1} \,E_{\text{m}}.$$

For further calculation of the *h* value and the reconciliation procedure, *R* has to be stripped of rows with zero singular values, otherwise the inverse of $\Upphi$ might not exist or be too close to singular, leading into unstable results. The singular values can be read from the matrix Σ acquired by singular value decomposition of *R*. The number of non-zero singular values in Σ determines the number of rows in conversion matrix Σ_conv_; which has 1 in the diagonal else 0 (Eqs. 27 and 28).

Singular value decomposition of *R*

27 $$ R = U\Upsigma \,V.$$

Calculation of the reduced redundancy matrix *R*

28 $$ R_{\text{red}} = \Upsigma_{\text{conv}} \Upsigma \Upsigma^{\text{T}} U^{\text{T}}.$$

Now the *h* value can be calculated and the rates reconciled as explained above by replacing *E* with *R*_red_ in Eq. (20). Finally the vector for estimated rates can be calculated according to Eq. (29), using the reconciled rates instead of raw measured rates. Furthermore, the estimated rates can be numerically integrated to estimate the cumulated biomass in the time window $\Updelta t$ according to Eq. (30), which is called cumulative elemental balancing further on.

Calculation of the estimated rate vector

29 $$ W_{\text{c}} = E_{\text{c}}^{ - 1} \,E_{\text{m}} \,W_{\text{b}}. $$

Estimation of the biomass by numeric integration of the items in the estimated rate vector $W_{\text{c}}$ (stoichiometric cumulation) Eq. (30)

30 $$ X = r_{{X\_{\text{recon}}}} *\Updelta t\, + \,X_{\text{initial}}. $$

## Results

Correct assumption of growth stoichiometry is an important prerequisite for the elemental balancing approach proposed in this contribution. By proper application of elemental balances products such as ethanol or acetate can be quantified [16]. However, this has to be addressed beforehand, by setting up the soft sensor accordingly. If the residuals on the elemental balances are higher than the defined error according to Ψ, faulty definition of stoichiometric growth is detected. This is evaluated by statistical test, which takes measurement error according to the Chi-square distribution into account. Hence, Ψ has to be defined based on a realistic assumption of the measurement error, as failure to do so will result in potentially misleading results and/or statistical test values (“Consistency check”).

There are processes which generally follow balanced growth conditions, which means metabolic or morphological variations due stress and fermentation conditions are negligibly small and do not propagate to coefficients in data-driven or mechanistic models. In that case the estimation problem is much easier, since coefficients are constant throughout the experiment and also for follow-up experiments (see “Induced culture without significant extracellular product”). However, this is not true for any kind of process (see “Induced culture with significant extracellular product and variable yields”); hence, poor model generalization can become a major issue. Here, the elemental balancing approach poses a valuable alternative.

### Induced culture without significant extracellular product

**Fig. 2:**
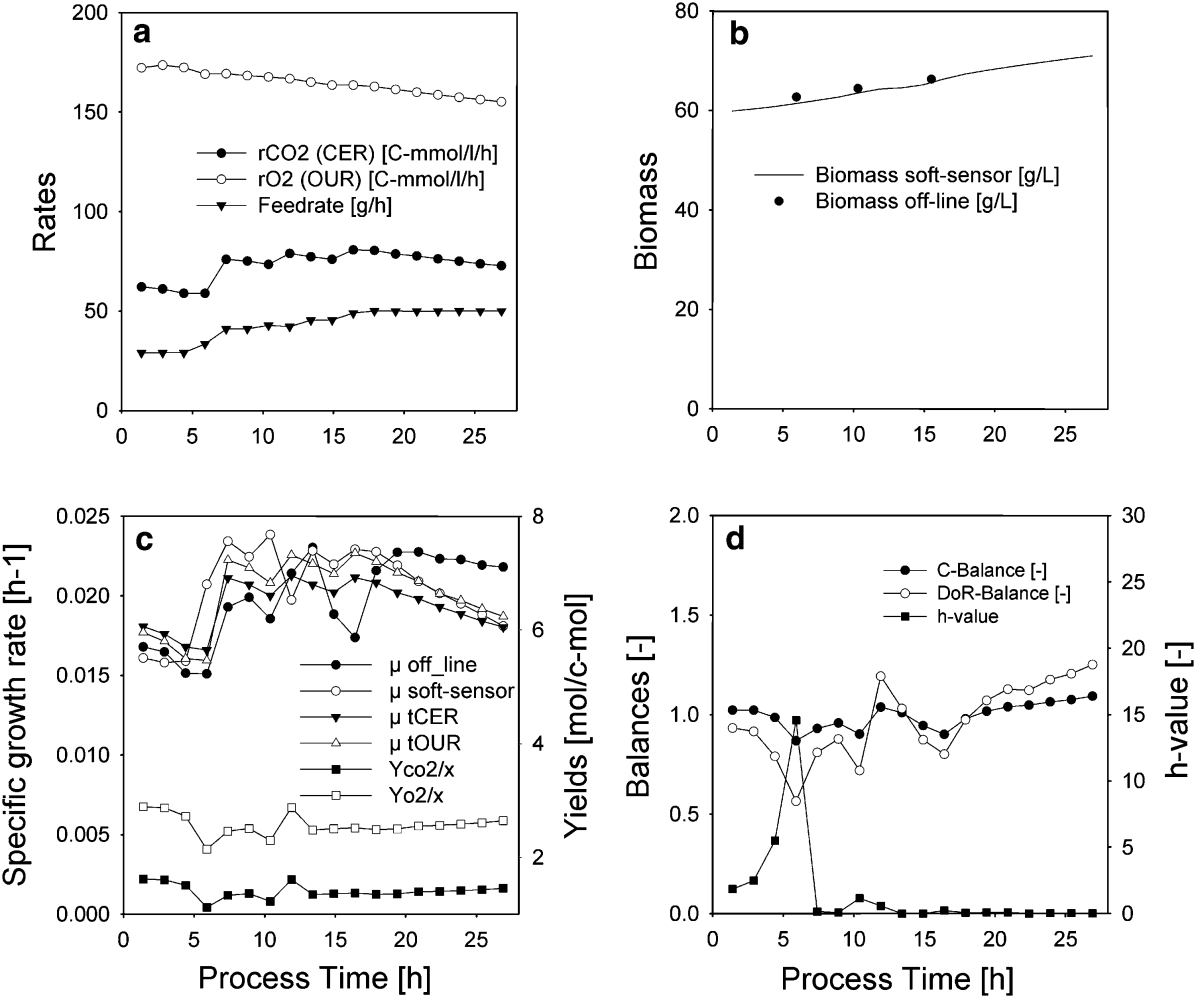
Induced *P. Pastoris* fedbatch on methanol without significant extracellular product **a** inputs; **b** measured biomass concentration and estimated biomass concentration by soft sensor based on cumulative elemental balancing; **c** specific growth rate calculated from interpolated offline biomass samples (μ off-line), off-gas rates (μ tCER and μ tOUR, together with the respective yields: Yco2/x and Yo2/x) and based on cumulative elemental balancing (μ soft-sensor); **d** elemental balances for off-line biomass concentrations and *h* value for soft sensor; between process time 5 and 10 h the *h* value and the balances were off, probably due to consumption of substrate accumulated during the onset of the methanol feeding

Data from an induced *P. pastoris* culture were used as an example for a culture without significant product (volumetric rate of substrate uptake is of magnitudes higher than the volumetric rate for extracellular product). Furthermore, this culture exhibits balanced growth conditions with regard to energy metabolism, since the yields are rather constant as also shown below (<10 % variation). This can probably be attributed to the careful adaption of the culture to the methanol feed and moderate expression rates due to the single-copy strain used in this process [30], which results in a constant maintenance part in the yields. A soft sensor for fed-batch with oxidative metabolism according to “Estimation” was implemented to estimate the biomass concentration in real-time from signals which are available on-line only. The input rates for this soft sensor were OUR, CER (measured by Off-gas analysis), the substrate feed rate and the reactor broth mass (by gravimetric principle, respectively) as shown in Fig. 2a. The output of the soft sensor was the biomass concentration (Fig. 2b) calculated using the cumulated biomass (Eq. 30) and current volume together with subsequently calculated variables such as the specific growth rate according to “Specific rates and yields”. Figure 2c shows the specific growth rate for calculated using different sources for the biomass quantification:

- *Off*-*line* Smoothed and interpolated off-line values according to Eq. (12) were used to calculate *μ* off-line (Fig. 2c). To check the quality of the off-line values, elemental balances such as the C and DoR balance can be imposed. If the law of conservation is satisfied the balance are close to 1, which is true here, if they deviate gross errors such as incorrectly assumed growth stoichiometry (e.g. oxidoreductive instead oxidative) or substrate accumulation are present (Fig. 2d).
- *tCER and tOUR* Estimation by off-gas rates only according to Eq. (15) was used to calculate *μ* *t*CER and *t*OUR. This method is only useful if the correlation of off-gas rates to biomass conversion rate is constant, or in other words the yields do not vary too much (only <10 %), which can be assumed for this culture (Fig. 2c). This can be probably attributed to the careful adaption of the culture to methanol and moderate foreign protein expression rates [28], which results in a constant maintenance part in the yields. This approach is comparable to similar methods without variable yields.
- *Soft sensor* Estimation of *μ* using cumulative elemental balancing as described in “Estimation” (Fig. 2c). This method allows flexible yields but the growth stoichiometry (e.g. oxidative metabolism) has to be correctly assumed beforehand. The *h* value is based on elemental balances (“Consistency check”) and is used as a real-time acceptance criterion. If the value is lower than the threshold value of 3.84, no gross errors are present and the growth stoichiometry was assumed correctly, which holds true for this culture (Fig. 2d).

Since the off-gas yields were rather constant (Fig. 2c) and the growth was purely oxidative, all methods give an estimate of the specific growth rate in good agreement with the values calculated from off-line biomass concentrations. Between process time 5 and 10 h the *h* value and the balances were off, probably due to consumption of substrate, which was accumulated during the onset of the methanol feeding.

### Induced culture with significant extracellular product and variable yields

**Fig. 3:**
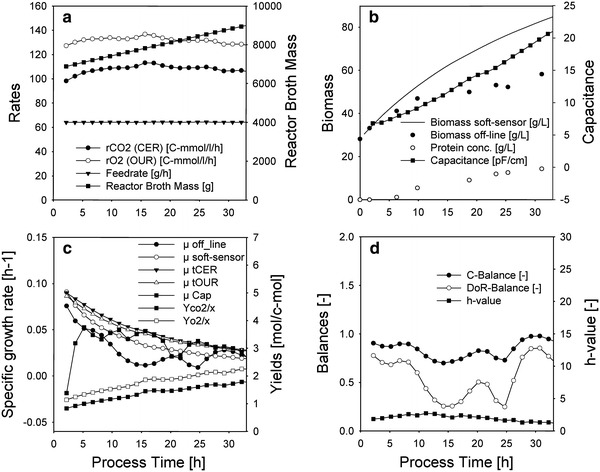
Induction phase of *E. coli* culture with significant product with *μ*_initial_ = 0.10 (h^−1^); **a** inputs for the soft sensor based on elemental balancing; **b** measured biomass concentration and estimated biomass concentration by soft sensor based on elemental balancing; **c** specific growth rate calculated from interpolated offline biomass samples (μ off-line), off-gas rates (μ tCER and μ tOUR, together with the respective yields: Yco2/x and Yo2/x), capcaitance probe (μ Cap) and based on cumulative elemental balancing (μ soft-sensor); **d** elemental balances for off-line biomass concentrations and *h* value for soft sensor

**Fig. 4:**
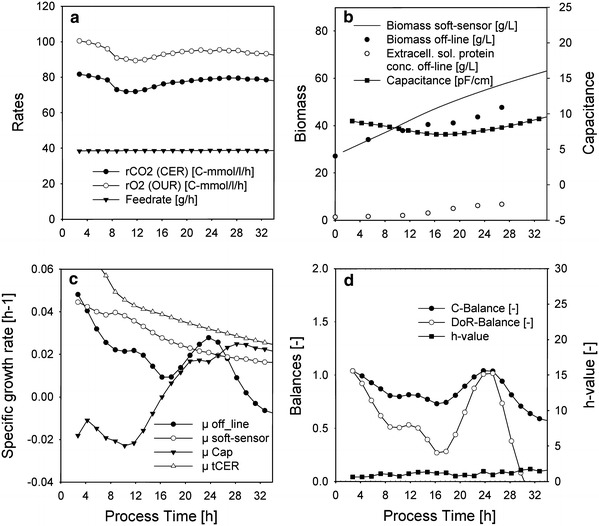
Induction phase of *E. coli* culture with significant product with *μ*_initial_ = 0.06 (h^−1^); **a** inputs for the soft sensor based on elemental balancing; **b** measured biomass concentration and estimated biomass concentration by soft sensor based on elemental balancing; **c** specific growth rate calculated from interpolated offline biomass samples (μ off-line), off-gas rates (μ tCER), capcaitance probe (μ Cap) and based on cumulative elemental balancing (μ soft-sensor) **d** elemental balances for off-line biomass concentrations and *h* value for soft sensor

The applicability of the soft-type sensor based on cumulative elemental balancing was evaluated by estimation of biomass and the specific growth rate in a culture with significant extracellular product and variable yields. Results were compared with a Luedeking–Piret-type approach, a capacitance probe (“Capacitance probe”) and conventional off-line sampling. Two different fed-batch experiments were evaluated. In one experiment the initial specific growth rate was adjusted from μ = 0.15 (h^−1^) to a *μ*_initial_ = 0.10 (h^−1^) (Fig. 3), while in the other experiment the initial specific growth rate was adjusted from *μ* = 0.15 (h^−1^) to a *μ*_initial_ = 0.06 (h^−1^) (Fig. 4).

Signals which are available on-line were used as input signals for the soft sensor (Figs. 3, 4a). The estimated biomass concentration deviates from the off-line biomass concentration progressively with process time (Figs. 3, 4b). If the extracellular soluble protein concentration is added to the biomass, which is justifiable since the stoichiometry is very similar, this deviation is smaller and the remainder can probably be attributed to extracellular soluble non-protein content. This is due to the fact that the elemental balancing approach estimates overall bioconversion, biomass and any other soluble building block material. By this approach, it is not possible to distinguish between extracellular products such as soluble protein and cellular biomass since both have very similar stoichiometry (compared to classical oxidoreductive products such as ethanol).

Capacitance is described as a powerful tool for recombinant *E. coli* processes in literature [38]. The probe quantifies capacitance in (pF/cm), which typically has to be converted to more common units such as biomass (g/L) or optical density (–) for further use (by means of linear regression). An important thing to note is that capacitance relates to intact biovolume [31] and not necessarily to biomass. Schwan’s model [31], which describes the relation between enclosed biovolume or capacitor volume and the dielectric increment, also suggests *C*_m_ [plasma membrane capacitance per unit of membrane area (F/m^2^)] as a parameter, which relates to the ability of the plasma membrane to store charge.

Schwan’s model Eq. (31)

31 $$ \Updelta C*\frac{k}{{\varepsilon_{0} }} = \Updelta \varepsilon^{'} = \frac{{9*P*r*C_{\text{m}} }}{{4*\varepsilon_{0} }}. $$

Figures 3 and 4c show the specific growth rate calculated using different sources of the biomass concentrations:

- *Off*-*line* Smoothed and interpolated off-line values according to Eq. (12) were used to calculate *μ* off-line. To check the consistency of the off-line values, elemental balances, namely a C- and a DoR balance were imposed. Figures 3 and 4d show that both balances are <1, which can be attributed to the extracellular soluble protein unaccounted for in this balance.
- *tCER and tOUR* Estimation by off-gas rates only according to Eq. (15) was used to calculate *μ* *t*CER and *t*OUR. Figure 3 and 4c show that the yields are increasing for this culture (up to 70 % increase over process time), hence the estimated specific growth rate is artificially larger than the other growth rates. If this method is used to estimate the specific growth without verification by other methods and lacking a strategy to account for the variable maintenance part of the yield in real-time, results are misleading due to the variable yield.
- *Soft sensor* Estimation using elemental balancing and reconciliation as described in “Data reconciliation” and “Estimation”. This method allows flexible yields but the growth stoichiometry (e.g. oxidative metabolism) has to be correctly assumed beforehand. The *h* value is based on elemental balances (“Consistency check”) and is used as a real-time acceptance criterion. If the value is lower than the threshold value of 3.84 no gross errors are present and the growth stoichiometry was assumed correctly. The value is below the threshold value (Figs. 3, 4d). The estimated specific growth rate is larger than the off-line specific growth rate due to the fact that this growth rate is based on the overall biomass conversion rate, including extracellular soluble components.
- *μ capacitance* The capacitance signal relates to biovolume and not to biomass, hence the linear regression model possibly also interferes with the parameter membrane capacitance as discussed above. There seems to be a dramatic decrease in biovolume due the reduction of specific growth rate from *μ* = 0.15 (h^−1^) to *μ*_initial_ = 0.1 (h^−1^) or 0.06 (h^−1^) at the onset of induction (Figs. 3, 4c). Such a decrease in volume due to carbon depletion was also reported in literature [39]. The parameter *C*_m_ in Schwan’s model (Eq. 31), which is in fact another morphological parameter, can also interfere with quantification. For this reason the apparent specific growth rate calculated from the capacitance signal is low or even negative during the initial hours of induction. This is even more pronounced in Fig. 4c, obviously since the drop in feed rate is larger here.

## Discussion

Different methods to estimate the biomass concentration and also the specific growth rate μ in real-time were compared. These variables are typically determined by time-consuming off-line sampling, which is also prone to operator-dependent measurement error. The biomass concentration is estimated from signals which are available on-line only. A real-time biomass concentration is required for real-time calculation of specific rates and yields, which in turn provide valuable information on the bioprocess.

Specific growth rates calculated using total carbon or oxygen rates can only be used as long as the respective yields are constant. This was true for the induced *P. Pastoris* culture (Sect. 3.1), but not for the *E. coli* culture (Sect. 3.2). Since the yields varied up to 70 %, estimated growth rates are artificially too large by this factor. Without verification by other means to estimate the biomass or a strategy to account for the variable maintenance part in the yields, the results are potentially misleading.

The soft sensor cannot differentiate between biomass and very similar soluble products such as protein in the supernatant, hence estimates an overall bioconversion rate (anabolic conversion) of substrate, instead of conversion to cells or protein. If the off-line extracellular protein is added to the off-line biomass, the result is similar to the estimation of the soft sensor. Potentially extracellular protein can be quantified in real-time if required, e.g. by at-line spectroscopic or photometric methods.

Provided balanced growth conditions, the cell volume and therefore the capacitance signal linearly correlates to viable biomass dry weight. In induced systems the assumption of balanced growth does not necessarily hold true, as shown in this contribution. If this is not considered, it can be misleading to use this as a sole method to quantify biomass. Along with possible variability in the ratio biovolume per biomass, variations of the parameter *C*_m_ would also interfere with linear regression models for the estimation of biomass. While this can be interesting additional information on the bioprocess, further off-line measurements of morphological characteristics such *E. coli* size distribution (e.g. by coulter principle) or radiofrequency scanning, as shown by other authors [40], are required to fully identify the source of variability. However, this was not within the focus of this contribution.

The quantification of biomass in the induction phase is not straight forward due to possibly variable cell metabolism, morphology and variables describing the former such as yields. There are multiple possible definitions for biomass. Conventionally biomass is defined as the non-soluble fraction of the culture broth after removal of water. It is not possible to apply this method in situ in real-time for obvious practical reasons. Furthermore, this definition might not be the best one from a physiological point of view, since there is more to biomass than just non-soluble mass. Elemental balancing for example allows quantifying an overall anabolic bioconversion, including soluble cell components secreted to the supernatant, such as carbohydrates and proteins. Capacitance provides information on the intact biovolume. If frequency scanning or additional off-line measurements are used, also other interesting morphological information can be revealed.

The biomass concentration, typically referred to as biomass dry cell weight concentration, is considered to be a key variable for the design of control strategies. This contribution outlined that there are multiple variables which describe (e.g.: biovolume, total bioconversion). Hence, the question arises on what measurement basis a control strategy, e.g. for the induction phase feed profile, should be used. Accordingly other authors suggest that the induced cell population is not homogeneous and segregated models need to be applied to account for different sub-populations [41]. In a control context, the question arises how to make different subpopulations quantitatively accessible by means of on-line and real-time sensors. This is also a question of what is the link of the biomass definition to product quality or productivity. One sensor might not suffice for this task, but combinations of different analytical devices in combination with mechanistic models/soft sensors, might be able to unlock the current status of the cell population.

## Conclusion

- A real-time capable soft sensor based on elemental balancing to quantify biomass concentration and specific growth rates was presented. Quantification of biomass in the induction phase is particularly challenging, due to the morphological, physiological and metabolic variations during induction. The presented method works with variable yields, which makes this approach especially interesting in the induced phase of recombinant bioprocesses, compared to other approaches with fixed yield or model coefficients as detailed in “Discussion”. As demonstrated, other approaches with fixed yield or model coefficients, such as the Luedeking–Piret-type approach and the linear regression model for the capacitance probe, have issues in the induction phase. Furthermore, it was shown that the reference method, gravimetric biomass dry weight determination, cannot quantitatively cover anabolic bioconversion, due to secretion of soluble contents to the supernatant.
- While the basic idea of elemental balancing, as a method to estimate unknown items such as the biomass concentration in a bioprocess, has been published decades earlier, the methodology is not used in modern bioprocess technology including recombinant bioprocesses so far. Furthermore, the approach is underrepresented in recent reviews [10, 42]. The ease of use and the low requirements on prior knowledge, as also requested by these reviews, make this method a particularly valuable tool for industrial bioprocesses.
- The results were compared and discussed with other state of the art approaches for real-time quantification of biomass. The optimum approach depends on the favored definition for “biomass” (conventional biomass, anabolic bioconversion, biovolume, etc.). Dependent on the process and/or product a definition with a clear link to product quality or productivity should be used.
- Since the results of this approach are obtained in real-time, this can be used to estimate process variables for control strategies. Specific rates such as the specific growth rate are especially important process variables, acting as a descriptor of the physiological state, with impact on biomass production, product quantity, and product quality [43]. Furthermore, if a system is fully quantified, a great deal of understanding is achieved that can be easily communicated.
- This approach can be applied for platform approaches, new trends toward platform manufacturing and decreases time to market.

## List of symbols

*t*: Time (h)
*S*: Total amount of substrate in the cultivation broth (C-mol)
$\dot{S}$: Substrate feed rate (C-mol/h)
*r*: Conversion rate (C-mol/h)
*q*: Specific rate (g/g/h)
*Y*: Yield (C-mol/C-mol)
*F*: Flow/feed rate (g/h) for liquid and (nL/min) for gas
*C*: Concentration (C-mol/l)
*X*: Total amount of biomass in the cultivation broth (C-mol) or (g)
*y*: Mole fraction (–)
*V*_m_: Molar volume of gas at norm condition (0 °C and 1 atm) (nl/mol)
Ra_inert_: Inert gas ratio (–)
y_wet_: O_2_ Conc. diluted by water content (without bioreaction) (–)
*N*: Total amount of ammonium in the cultivation broth (mol)
O_2_: Total amount of oxygen in the cultivation broth (mol)
*Z*_*i*_: Elemental composition of component *i* in biomass (–)
*p*_*i*_: Elemental composition of component *i* in substrate (–)
*V*: Volume of the cultivation broth (l)
${\text{ex}}_{{{\text{H}}_{2} {\text{O}}}}$: Water content in off-gas (–)
*M*: Molecular weight (g/c-mol)
*ρ*_feed_: Density of feed (g/L)
*S*_0_: Feed concentration (g/L)
*γ*: Degree of reduction (–)
*ν*: Coefficients e.g. ν (–)
OD: Optical density 600 nm (–)
*k*: Specific growth rate if used for feed rate calculations (h^−1^)
*μ*: Measured specific growth rate (h^−1^)
CER: Carbon dioxide evolution rate (C-mol/h)
OUR: Oxygen uptake rate (C-mol/h)

## Indices

in: Input
out: Output
conv.: Conversion
acc.: Accumulation
*s*: Substrate
*f*: Feed
*x*: Biomass
*a*: Air
CO_2_: Carbon dioxide
O_2_: Oxygen
N: Ammonium
b: Base
O: Oxygen
H: Hydrogen
n: Nitrogen
*m*: Measured
*c*: Estimated
*i*: Item number *i*
*j*: Item number *j*
*t*: Time point *t*
0: Initial
